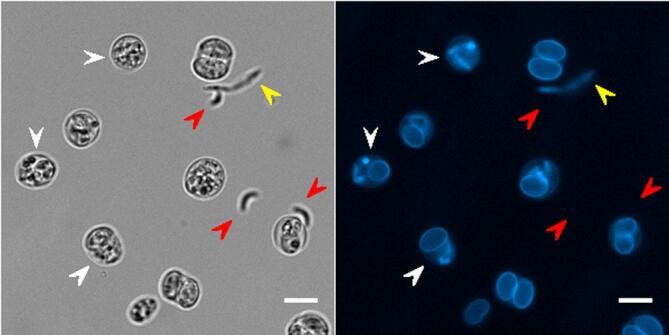# Structure, composition, and roles of the *Toxoplasma gondii* oocyst and sporocyst walls

**DOI:** 10.1016/j.tcsw.2018.100016

**Published:** 2018-12-19

**Authors:** Wesley Freppel, David J.P. Ferguson, Karen Shapiro, Jitender P. Dubey, Pierre-Henri Puech, Aurélien Dumètre

**Affiliations:** a Aix Marseille Univ, IRD, AP-HM, SSA, VITROME, Marseille, France; b Nuffield Department of Clinical Laboratory Science, University of Oxford, John Radcliffe Hospital, Oxford OX3 9DU, United Kingdom; c Department Biological & Medical Sciences, Oxford Brookes University, Oxford OX3 0BP, United Kingdom; d Department of Pathology, Microbiology & Immunology, School of Veterinary Medicine, One Shields Ave, 4206 VM3A, University of California, Davis, CA 95616-5270, USA; e United States Department of Agriculture, Agricultural Research Service, Beltsville Agricultural Research Center, Animal Parasitic Diseases Laboratory, Building 1001, Beltsville, MD 20705-2350, USA; f Aix Marseille Univ, LAI UM 61, Marseille F-13288, France; g Inserm, UMR_S 1067, Marseille F-13288, France; h CNRS, UMR 7333, Marseille F-13288, France

**Keywords:** Oocyst, Sporocyst, Wall structure, Molecules, Environment

## Abstract

*Toxoplasma gondii* is a coccidian parasite with the cat as its definitive host but any warm-blooded animal, including humans, may act as intermediate hosts. It has a worldwide distribution where it may cause acute and chronic toxoplasmosis. Infection can result from ingestion either of tissue cysts in infected meat of intermediate hosts or oocysts found in cat faeces via contaminated water or food. In this review, we highlight how the oocyst and sporocyst walls sustain the persistence and transmission of infective *T. gondii* parasites from terrestrial and aquatic environments to the host. We further discuss why targeting the oocyst wall structure and molecules may reduce the burden of foodborne and waterborne *T. gondii* infections.

## Introduction

**Fig. 1:**
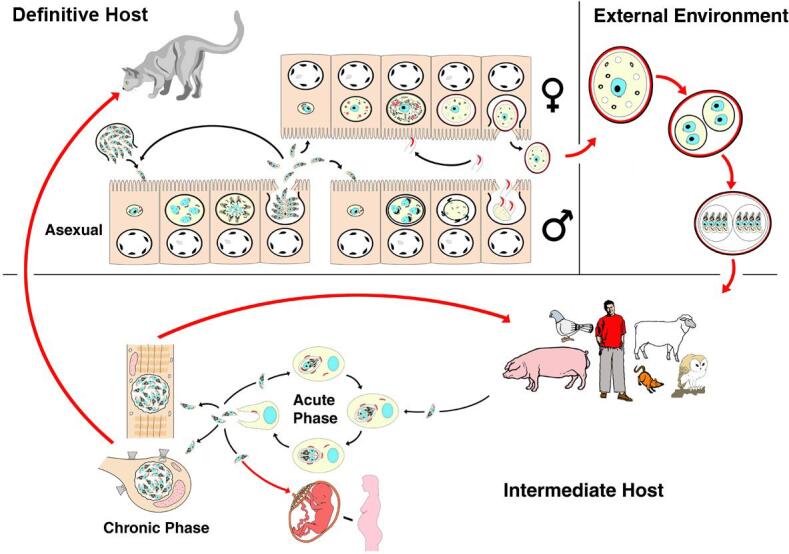
Diagrammatical representation of the life cycle of *Toxoplasma gondii* showing the development in the definitive and intermediate hosts plus oocyst sporulation in the external environment. Red arrows show transmission between hosts while black arrows represent development within the host. From Ferguson (2002) with permission.

*Toxoplasma gondii* is a coccidian parasite with the cat as its definitive host, but any warm-blooded animal, including humans, can act as intermediate hosts. It has a worldwide distribution with approximately 30% of the human population infected although this varies markedly between populations (Dubey, 2010). While the vast majority of infections are asymptomatic, the parasite can cause serious disease to the foetus in women first infected while pregnant (congenital transmission) or in people with a compromised immune system (AIDS, cancer and transplant patients) (Robert-Gangneux and Dardé, 2012). It has a complex life cycle in which sexual phases are limited to the definitive host (felids) while asexual phases can occur in both the definitive and intermediate hosts (Fig. 1). Sexual reproduction results in formation of environmentally resistant oocysts that are excreted exclusively in faeces of felids (VanWormer et al., 2013). People are infected either by eating vegetables, fruits, and water contaminated with oocysts or raw/undercooked meat containing tissue cysts of the parasite. There is little evidence that the route of infection plays a role in subsequent disease in humans with a normal immune system being the major factor in controlling disease/infection. However, there is some evidence that specific symptomatic outbreaks were associated with oocyst contamination (Teutsch et al., 1979, Stagno et al., 1980, Benenson et al., 1982, Bowie et al., 1997, de Moura et al., 2006, Palanisamy et al., 2006) but this may be misleading since it is unlikely that a single meat source (tissue cyst sample) will infect a cohort of unrelated individuals. As humans are omnivores, they can be infected by either route but oocyst will play a very important role in the infection of herbivores, which can then act as a source of human infection. The importance of the oocyst in maintaining *Toxoplasma* infections was also suggested by early studies showing that *Toxoplasma* infection was absent in islands without cats (Wallace et al., 1972, Dubey et al., 1997a). Treatment regimes for acute infections or recrudescence of chronic infection are restricted to few specific drugs and a human vaccine is lacking (Robert-Gangneux and Dardé, 2012). There is no drug that can eliminate the tissue cysts meaning that chronic infections cannot be eliminated thus leaving the host susceptible to recrudescence. Preventing the transmission of the parasite, in particular via the environmentally resistant oocysts, is therefore essential to reduce the burden of the disease worldwide.

While transmission to humans via tissue cysts is relatively easy to control via good kitchen hygiene and proper cooking of meat, oocysts are difficult to inactivate or remove from contaminated soils, waters and foodstuffs. Indeed, oocysts can survive for months, possibly years, in moist soils and fresh and marine waters under various temperatures (−20 to +37 °C) (reviewed by Dumètre and Dardé, 2003) and salinity conditions up to 15 ppt (*parts per thousand*) (Lindsay et al., 2003), allowing them to be transmitted to numerous host species living in different terrestrial and aquatic environments, including marine biotopes (VanWormer et al., 2013). Only higher temperatures (>45 °C) and desiccation can decrease oocyst viability in natural settings (Yilmaz and Hopkins, 1972, Dubey, 1998, Lélu et al., 2012). Moreover, oocysts are resistant to chemical inactivation agents, especially strong acids, detergents, and disinfectants such as household bleach or gaseous chlorine and ozone treatments used by the water industry (see Jones and Dubey, 2010 for review). In this regard, oocysts can pose important health hazards in areas where drinking water systems are solely based on chemical disinfection and not filtration or in cases of water treatment plant dysfunction (Jones and Dubey, 2010). Chlorinated aqueous solutions could also be ineffective in killing the oocysts in minimally processed vegetables (e.g. ready-to-eat packaged salads) as suggested recently (Hohweyer et al., 2016). Oocysts in suspensions can also resist physical inactivation by ultraviolet (UV) irradiation up to very high doses (>500 mJ/cm^2^) (Wainwright et al., 2007a). Actually, only heating above 60 °C can lead to the rapid killing of the oocysts (Jones and Dubey, 2010).

It is yet unclear how *T. gondii* oocysts can survive so many and varied harsh conditions. Though the sporozoites could rely on their own defence mechanisms to overcome some external stresses (e.g. UV, desiccation, high salinity) (Fritz et al., 2012a, Possenti et al., 2013), the oocyst and sporocyst walls are the key structures that provide mechanical and chemical protection to the sporozoites until their release in the host digestive tract (Christie et al., 1978, Ferguson et al., 1979d, Fritz et al., 2012a, Dumètre et al., 2013). In the light of available structural and molecular studies on *T. gondii* oocysts and related coccidian species, we review how the oocyst walls secure the transport of the oocysts from the environment to the host and discuss why targeting these walls could be critical for control of *T. gondii* infections in humans and animals.

## Formation and structure of the oocyst and sporocyst walls

Oocysts result from the sexual multiplication of the parasite in the small intestine of cats (Hutchison et al., 1969, Frenkel et al., 1970, Sheffield and Melton, 1970). The cat is normally infected by ingesting tissue cysts present in the tissue of a chronically infected intermediate host (rodent/bird). The parasites (bradyzoites), released in the intestine, invade the enterocytes and initiate coccidian development, which involves several rounds of asexual replication prior to differentiating into “female” macrogametes or flagellated (motile) “male” microgametes (Fig. 1) (Ferguson, 2002). However, while cats can also be infected by ingestion of oocysts, the sporozoites do not appear to be able to directly initiate coccidian development as evidence by the extended pre-patent period (Dubey, 2006).

**Fig. 2:**
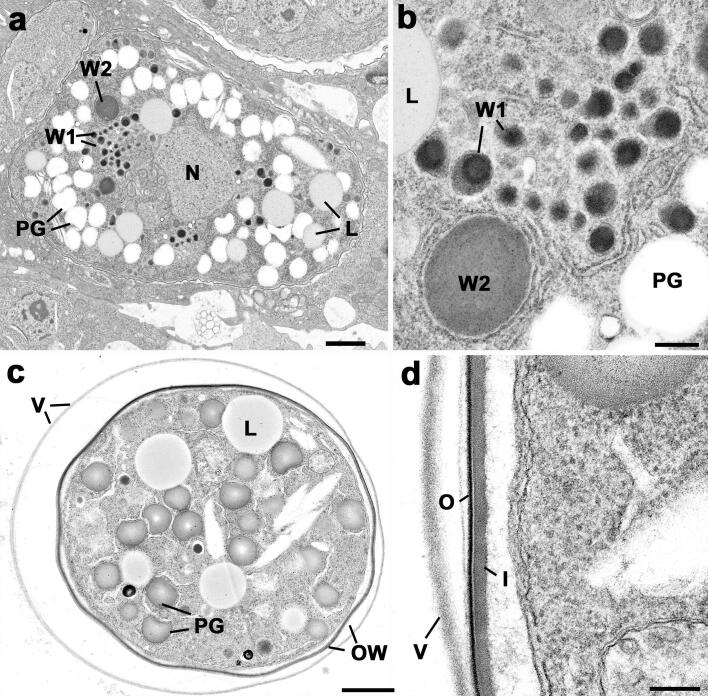
Electron micrographs of the macrogamete and early oocyst in the cat gut. (a) Mature macrogamete showing the central nucleus and the cytoplasm containing a number of wall forming bodies type 1 (W1) and 2 (W2) plus polysaccharide granules (PG) and lipid droplets (L). Bar = 1 µm. (b) Detail of the cytoplasm showing the W1 and W2 retained with the rough endoplasmic reticulum. L – lipid droplet; PG – polysaccharide granule. Bar = 100 nm. (c) Section through an oocyst showing the outer veil (V) and oocyst wall (OW) and the central cytoplasmic mass containing lipid droplets (L) and polysaccharide granules (PG). Bar = 1 µm. (d) Detail of the periphery of the oocyst showing loose veil (V) and the oocyst wall consisting of a thin electron dense outer layer (O) and thicker more electron lucent inner layer (I). Bar = 100 nm. The techniques used and images are from Ferguson, 2009, Ferguson et al., 2000 with permission.

Mature macrogametes contain numerous polysaccharide granules, lipid droplets and wall-forming bodies type 1 (W1) and 2 (W2) (Ferguson et al., 1975) (Fig. 2a and b). The W1 can, by immune-electron microscopy, be divided into two subtypes. W1subtype a (W1a, also called VFB for veil-forming bodies) are 350-nm electron-dense vesicles that locate at the periphery of the macrogamete which give rise to an outer veil (Ferguson et al., 2000). When similar structures were identified in *E. maxima* the term veil forming bodies was proposed (Ferguson et al., 2003) and will be used throughout this paper. In *T. gondii*, the W1b (subsequently will be termed W1) are a similar size but have more heterogeneous contents and a central position (Fig. 2a). The W2 are larger (∼1.2 µm in diameter), less numerous and more electron-lucent than W1 subtypes and are retained within the rough endoplasmic reticulum (Fig. 2b). It is believed that, after fertilization, there is the sequential secretion of the veil and wall forming bodies to form the oocyst wall, as described for parasites of the closely related *Eimeria* genus (Ferguson et al., 2003, Belli et al., 2006, Belli et al., 2009). The material coming from the VFB is secreted first and accumulates beyond the macrogamete limiting membrane to form a loose veil-like structure (Fig. 2c and d) (Ferguson et al., 1975, Ferguson et al., 2000, Belli et al., 2006) The oocyst wall *per se* develops underneath the veil. The outer layer is synthesized first by the secretion of W1 and forms an ∼20 nm thick electron dense layer (Fig. 2d). This is followed by the secretion of the W2 to form the more electron-lucent and thicker (∼30 to 70 nm) inner layer (Fig. 2d). Oocysts exit from the enterocytes and pass in cat faeces often in huge quantities numbers (>10^7^ oocysts/cat for 7–15 days) even following oral infection by a single parasite (Dubey, 2001).

**Fig. 3:**
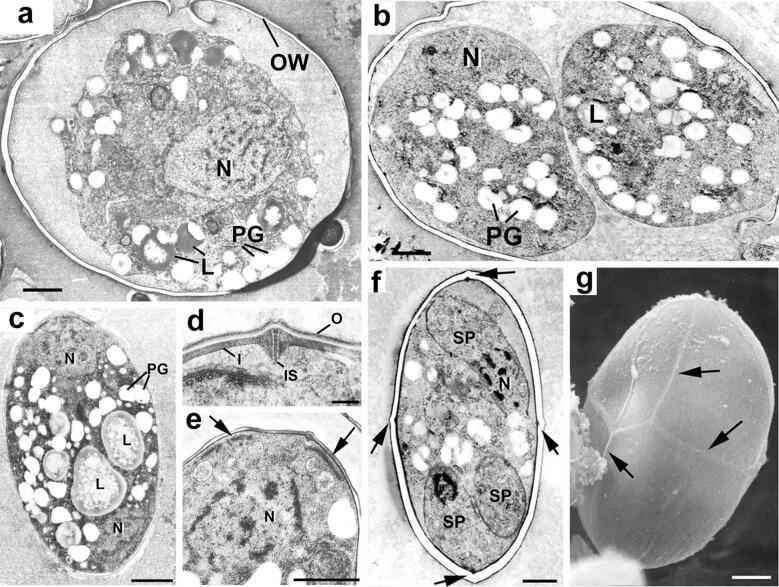
Electron micrographs illustrating changes occurring during sporulation. (a) Section through an unsporulated oocyst (zero hour) showing the central nucleus (N) with cytoplasm containing a number of polysaccharide granules (PG) and lipid droplets (L). Bar = 1 µm. (b) Section through the two secondary sporoblasts. N, nucleus; L, lipid droplet; PG, polysaccharide granule. Bar = 1 µm. (c) Early development of the sporocyst showing the elongated appearance with a nucleus (N) located at either end of the sporocyst and the cytoplasm containing polysaccharide granules (PG) and lipid droplets (L). Bar = 1 µm. (d) Cross-section through the sporocyst wall which consists of a thin, continuous outer layer and an inner layer consisting of four plates. There is a swelling of the plates of the inner layer at the junction where they are joined by an intermediate strip (IS) of material. Bar = 100 nm.(e) Enlargement of part of a sporoblast showing the nucleus (N) and the two dense plaques (arrows) representing the initiation of daughter formation. Bar = 1 µm. (f) Advanced stage of sporozoite formation (SP) showing the nucleus becoming enclosed by the inner membrane complex of the daughters. The junction between the four plates of the sporocyst wall can be seen (arrows). Bar = 1 µm. (g) Scanning electron micrograph illustrating the raised junctions between the plates (arrows). Bar = 1 µm. The techniques used and images are from Ferguson et al., 1978, Ferguson et al., 1979a, Ferguson et al., 1979b, Ferguson et al., 1979c with permission.

**Fig. 4:**
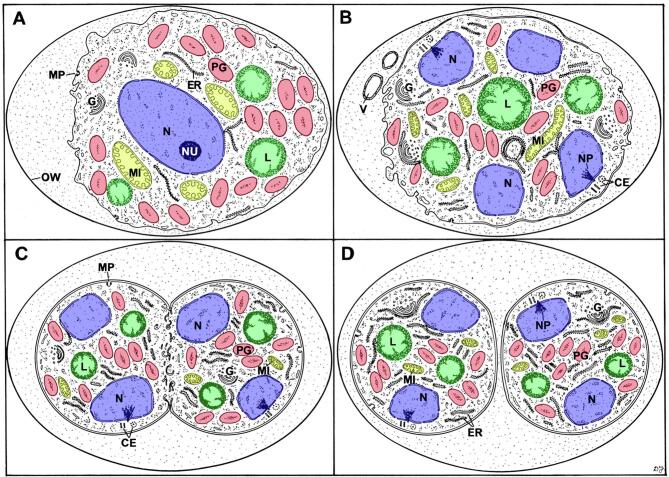
Diagrammatical illustration of the changes observed during early stages of sporulation. (A) An unsporulated oocyst (zero hour). (B) A sporulating oocyst; (C) Formation of the sporoblasts; (D) Early sporoblasts. CE = centriole; ER = rough endoplasmic reticulum; G = Golgi body; L = lipid droplets (green); MI = mitochondrion (yellow); MP = micropore; N = nucleus (blue); NP = nuclear pole; NU = nucleolus; OW = oocyst wall; PG = polysaccharide granule (red); V = vacuole. From Ferguson et al. (1979a) with permission.(For interpretation of the references to colour in this figure legend, the reader is referred to the web version of this article.)

**Fig. 5:**
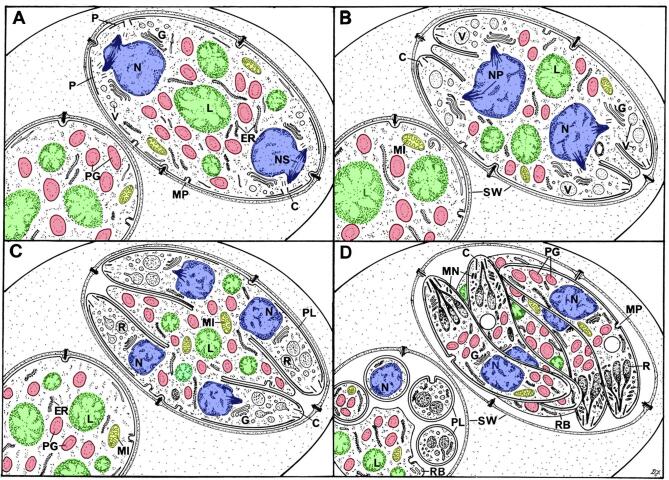
Diagrammatical illustration of the structural changes associated with the later stages in sporulation (A-D). C = conoid; ER = rough endoplasmic reticulum; G = Golgi body; L = lipid droplets (green); MI = mitochondrion (yellow); MN = microneme; MP = micropore; N = nucleus (blue); NP = nuclear pole; NS = nuclear spindle; OW = oocyst wall; P = plaque; PG = polysaccharide granule (red); PL = pellicle; R = rhoptry; RB = residual body; SW = sporocyst wall; V = vacuole. From Ferguson et al. (1979c) with permission. (For interpretation of the references to colour in this figure legend, the reader is referred to the web version of this article.)

Freshly excreted oocysts are ovoid and measure 10 × 12 µm. They contain a diploid cytoplasmic mass with numerous lipid droplets and polysaccharides granules surrounded by one or two unit membranes (Ferguson et al., 1979a, Ferguson et al., 2000). The veil is generally absent as it probably disintegrates upon oocyst shedding (Speer et al., 1998, Ferguson et al., 2000). At this point the cytoplasmic mass is isolated within the protection of the oocyst wall (Fig. 3a) and is unable to obtain any further nutrients thus the building blocks for all structural changes occurring during sporulation (sporocyst and sporozoite formation) must already be stored within the oocyst. In the environment, oocysts undergo further aerobic development (sporulation) to become infectious sporulated oocysts (Dubey et al., 1970a, Dubey et al., 1970b). This process usually takes 7 days at 20–25 °C and leads to the formation of two sporocysts, each hosting four sporozoites. Initially the newly secreted oocyst contains a single cytoplasmic mass; the primary sporoblast (Fig. 3a). The exact process and timing of the nuclear divisions within the primary sporoblast is still not completely understood (Ferguson et al., 1979a, Ferguson et al., 1979b, Dubey et al., 2017). However, it would appear that a meiotic (2N–N) and a mitotic division give rise to four haploid nuclei. There is division of the primary sporoblast by invagination of the limiting unit membranes to form two spherical secondary sporoblasts each with two nuclei (Fig. 3b). This process is summarised in Fig. 4. As the sporulation progresses, the sporoblasts elongate to form ellipsoidal sporocysts measuring 6 × 8 µm (Ferguson et al., 1979c). During this development, there is secretion of material to form the wall of the sporocyst that consists of an outer electron-dense layer (15–20 nm) that derives from the sporoblast membranes and an inner electron-lucent layer (40–50 nm) that appears to be synthesized de novo (Fig. 3c and d). The inner sporocyst wall layer is composed of four curved plates held together by thick sutures (Fig. 3d, f, g) (Ferguson et al., 1978, Ferguson et al., 1979b, Ferguson et al., 1979d, Ferguson et al., 1982, Speer et al., 1998). Each suture consists of an interposing strip connecting two adjacent plates by two thin electron-dense bands (Fig. 3d). In contrast to some coccidian species of the genus *Eimeria*, the sporocyst wall of *T. gondii* does not possess a plug-like structure such as a Stieda body (Berto et al., 2014). Each sporocyst possesses a nucleus at either end and two daughters form at the plasmalemma associated with each nucleus (Fig. 3c and e). Unlike other forms of asexual development in *T. gondii* where daughters form by budding within the cytoplasm, sporozoite formation appears as a budding from the plasmalemma (as described for most Coccidia). This results in the formation of four haploid crescent-shaped sporozoites measuring 2–6 × 8 µm in size and a residual mass containing polysaccharide granules and lipid droplets (Ferguson et al., 1979c, Ferguson et al., 1979d, Speer et al., 1998). This process is summarised in Fig. 5. The structure of the oocyst wall is retained throughout the sporulation, however it appears somewhat flexible as the oocyst becomes ellipsoidal and slightly increases in size (11 × 13 µm). The wall of sporulated oocysts may harbour a micropyle that appears as a small depression randomly located at its surface (Speer et al., 1998). The micropyle is thought to be a CO_2_-sensitive structure as described in several *Eimeria* species (Jensen et al., 1976). Its putative role in the sporozoite excystation is further discussed below.

## Molecular composition of the oocyst and sporocyst walls

Most of our knowledge on the molecules composing the *T. gondii* oocyst walls comes from pioneering works on the oocyst walls of the related *Eimeria* species and *Cryptosporidium parvum*.

### Proteins

As earlier reported in *E. maxima* and *E. tenella*, proteins appear as the most abundant (≥90%) components of the *T. gondii* oocyst walls (Mai et al., 2009). Global proteomic analyses of *T. gondii* oocysts have identified ∼225 proteins in oocyst wall fractions (Fritz et al., 2012a, Possenti et al., 2013, Zhou et al., 2017), with an abundance of proteins enriched in cysteine or tyrosine, and PAN-domain containing proteins. Few of them have been further characterized biochemically and assigned to one or both layers of the oocyst and/or sporocyst walls (SI Table 1).

A first set of oocyst wall proteins include cysteine- and histidine-rich oocyst wall proteins (OWP) that were first identified and characterized in *C. parvum* (COWP) (Spano et al., 1997, Templeton et al., 2004) and then in *T. gondii* (TgOWP). Of 12 TgOWP identified in whole oocysts or wall fractions, the TgOWP1 (homolog to COWP6), TgOWP2-3, and 8 were further characterized and found to locate exclusively in the outer layer of the oocyst wall (Templeton et al., 2004, Possenti et al., 2010, Salman et al., 2017). In contrast, COWP1 and COWP8 occur inside the oocyst wall of *C. parvum* (Spano et al., 1997, Templeton et al., 2004). OWP are prone to form complex polymers through disulfide bonds involving their cysteine residues in order to stabilize the overall structure of the oocyst wall as described for COWP1, a structural homolog of TgOWP1-7 in *C. parvum* oocysts (Spano et al., 1997). The synthesis and the mechanisms by which these proteins are incorporated in the developing walls of *T. gondii* oocysts are currently unknown. Investigations in *C. parvum* indicate that COWP1 is present inside wall-forming bodies of mature macrogametes prior its incorporation in the developing oocyst wall inner layer (Spano et al., 1997). We are not aware of any attempts to locate TgOWP in macrogametes/early oocysts, in particular in W1, which are associated to the formation of the outer oocyst wall layer. In addition, Walker et al. (2015) identified a hypothetical OWP protein in *E. tenella* (EtHOWP1) that locates to W2 in macrogametes and the inner oocyst wall layer. A homologue (TgHOWP1) was identified in mature *T. gondii* macrogametes (Walker et al., 2015) and oocysts (Fritz et al., 2012b), however its association to any of the oocyst wall layers has not been investigated. It is yet unclear if some OWP contribute as well to the *T. gondii* sporocyst wall formation as described for EnOWP2 and EnOWP6 proteins in the rodent *Eimeria nieschulzi* (Jonscher et al., 2015). Previous works on *T. gondii* are contradictory as Possenti et al. (2010) failed to detect TgOWP2 in the sporocyst wall while Fritz et al. (2012a) identified both TgOWP2 and TgOWP6 in the proteome of sporocyst/sporozoite fractions. As the oocyst walls may be difficult to finely separate from the sporocysts, OWP proteins in sporocyst fraction could be contaminants from the oocyst wall (Fritz et al., 2012a).

**Fig. 6:**
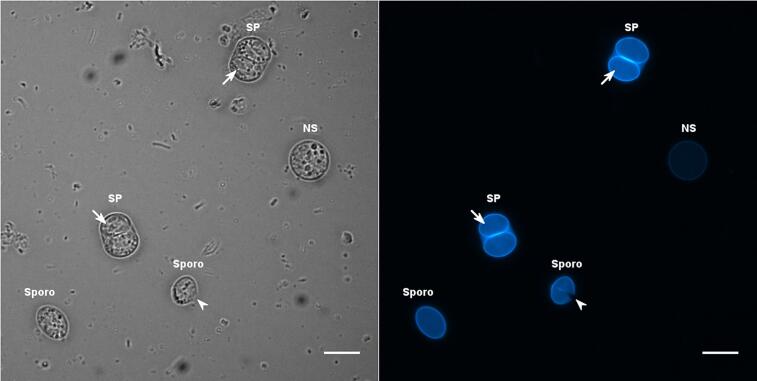
Oocysts and sporocysts of *Toxoplasma gondii*. This photomicrograph shows a spherical non sporulated (NS) oocyst filled with a single granular mass, two fully sporulated (SP) oocysts containing ellipsoidal sporocysts in which several banana-shaped sporozoites are visible (small arrow), and two individual sporocysts (Sporo). Note that the wall of one of these sporocysts is slightly open along one of its suture lines (arrowhead). The parasites were observed under bright field (left) or UV excitation for recording the autofluorescence pattern of the oocyst and sporocyst walls (right). Note the presence of faecal contaminants on the bright field image. Scale bar = 10 μm.

Tyrosine-rich proteins are also major components of the oocyst walls. They were first characterized in *E. maxima* where they locate to W2 in macrogametes and the inner oocyst wall layer (Ferguson et al., 2003, Belli et al., 2006, Mai et al., 2009, Walker et al., 2013). They appear to be very conserved across different *Eimeria* species (Krücken et al., 2008, Belli et al., 2009). These proteins, stored as proproteins in W2, are processed into smaller polypeptides by subtilisins prior to be incorporated to the developing oocyst wall. They form dityrosine cross-links involving their tyrosine residues following a process catalyzed by oocyst wall enzymes including an oxidase and several peroxidases (Mai et al., 2011, Walker et al., 2015). The dityrosine cross-links are thought to provide the structural robustness and lead to autofluorescence of the coccidian oocyst and sporocyst walls when visualized under UV irradiation (by using the DAPI-like filter set), as seen in *T. gondii* (Fig. 6). In-depth transcriptomic and proteomic analyses indicate that *T. gondii* oocysts contain *E. maxima*-unrelated Tyr-rich proteins, which appear to compose both layers of the *T. gondii* oocyst and sporocyst walls (Fritz et al., 2012a, Fritz et al., 2012b, Zhou et al., 2017). One of them (TyRP1) has been further characterized and found to locate in the outer oocyst wall (Fritz and Conrad, 2018), together with several enzymes required for dityrosine cross-linking as an oxidase (TgAO2, an homologue of EtAO2) (Walker et al., 2015) and aromatic amino acid hydroxylases that catalyze the conversion of tyrosine residues of wall proteins into 3,4-dihydroxyphenylalanine (DOPA) residues as a source of dityrosine (Belli et al., 2003, Wang et al., 2017). The sporocyst wall, which is also blue autofluorescent, could contain similar tyrosine-rich proteins and the dedicated enzymes for generating dityrosine cross-links. Interestingly, Walker et al. (2016) identified EtSWP1, a Tyr-rich protein specific to the sporocyst wall of *E. tenella*, but failed to detect any homologue in *T. gondii*.

In addition to TgOWP and Tyr-rich proteins, two putative PAN-domain containing proteins were identified as abundant components of the outer oocyst wall layer of *T. gondii,* but remain to be fully characterized (Fritz et al., 2012a, Possenti et al., 2013, Zhou et al., 2017). Such proteins could contribute in maintaining the wall architecture given their ability to form disulphide bridges. Other proteins including histidine and proline-rich proteins have been identified in the inner oocyst wall layer of *E. tenella* (Krücken et al., 2008). These proteins could contribute in stabilizing the wall of *E. tenella* oocysts together with Tyr-rich proteins. His/Pro-rich proteins have not been identified in *T. gondii* oocyst walls.

### Lipids

There are relatively small amounts (1–7%) of lipids in the oocyst walls of the coccidian parasites (Mai et al., 2009). In *T. gondii*, *E. maxima* and *E. tenella*, lipids are found in the outer layer of the oocyst and sporocyst walls mainly as cholesterol and acid-fast lipids with polyhydroxy fatty acyl chains (Mai et al., 2009, Bushkin et al., 2013, Frölich et al., 2013, Samuelson et al., 2013). Similar triglycerides have been described composing the outer layer of the wall of *C. parvum* oocysts lacking glycocalyx at their surface (Bushkin et al., 2013). In *E. maxima*, wall lipids originate from W1 found in mature macrogametes prior their incorporation into the developing outer oocyst wall layer. Some of the triglycerides identified in *E. tenella* oocyst walls resemble mycobacterial mycolic acids or lipids present in plant cutin (Bushkin et al., 2013). Consistent with this, polyketide synthases similar to those that make mycobacterial wall lipids have been detected in abundance in *T. gondii* and *E. tenella* oocysts (Bushkin et al., 2013).

In all these species, lipid coating would reinforce the robustness of the oocyst wall facing harsh external conditions and contribute to the impermeability of the coccidian oocysts to water-soluble molecules, including disinfectants and detergents. In contrast, oocyst wall lipids are very sensitive to treatments by polar molecules such as sodium hydroxide and organic solvents like chloroform/methanol, resulting in the loss of the outermost wall layer (Bushkin et al., 2013).

### Carbohydrates

In contrast to the *C. parvum* oocyst wall surface that is decorated by a glucose-rich glycocalyx (Jenkins et al., 2010), carbohydrates have appeared as minor components of the coccidian oocyst wall (≤2%). Mai et al. (2009) identified mainly galactose and glucose in the walls of *E. maxima* and *E. tenella* oocysts respectively. More recent investigations using the dectin-1 lectin has evidenced the presence of the fungal molecular attributes β-1,3 glucans within the inner oocyst wall layer of *E. tenella* and *T. gondii* (Bushkin et al., 2012, Samuelson et al., 2013). β-1,3 glucans arrange in fibrils to form a trabecular scaffold in the inner oocyst wall layer (Bushkin et al., 2012). This process appears to involve a glucan synthase that locates to the inner layer in *E. tenella* oocyst wall. However it is currently unknown how such sugar polymers are incorporated in the nascent oocyst wall regarding the sequential migration, disassembly and fusion of the different subsets of wall-forming bodies to the different layers of the coccidian oocyst wall.

It has been proposed that this β-1,3 glucan scaffold has a structural role by supporting the outer oocyst wall layer (Bushkin et al., 2012). Other sugar-containing molecules such as GalNAc and O-fucose-rich glycans have been identified in the inner oocyst wall layer and/or sporocyst wall of *T. gondii* by using the lectins *Maclura pomifera,* and *Ulex europaeus* 1 and *Aleuria aurantia* respectively (Samuelson et al., 2013, Bandini et al., 2016). Expanding the use of other lectins could allow the identification of other sugar-containing molecules in both the oocyst and sporocyst walls as described recently (Samuelson et al., 2013, Harito et al., 2016) (SI Table 1).

### Model for oocyst and sporocyst wall molecular composition

Overall, the current model for the oocyst and sporocyst wall composition of *T. gondii* and related coccidian parasites indicates that both walls are mainly proteinaceous with a lipid coating, making them very robust and hermetic to water-soluble molecules. The cross-linked Tyr-rich proteins appear to form the core structure of these walls. At the interface with the environment, the structure of the oocyst wall is reinforced by the addition of Cys-rich proteins and β-1,3 glucans in its outer and inner layers respectively. Many of these molecules are found in very different living organisms other than Coccidia such as plants, mycobacteria, fungi, and invertebrates (Samuelson et al., 2013), highlighting common strategies across kingdoms for resisting harsh environmental conditions. Functional characterization of the *T. gondii* oocyst wall components remains challenging as there is currently no in vitro system for producing large numbers of highly purified oocysts that would greatly facilitate genetic and biochemical investigations on this parasite stage.

## Roles of the oocyst and sporocyst walls

### In the resistance of the parasite to environmental insults and inactivating agents

The main deleterious conditions the parasite can encounter throughout the environment include variations in hygrometry, salinity, pH, temperature, solar radiation, and exposure to inactivating agents such as disinfectants (VanWormer et al., 2013). Oocysts are known to remain infective under some of these conditions for several days to several years (reviewed by Dumètre and Dardé, 2003, Jones and Dubey, 2010). This resistance has been attributed to the capacity of the oocyst and sporocyst walls to retain their integrity to some degree following chemical and physical stressors.

The protective role of the oocyst wall has been demonstrated in oocysts exposed to acids or chlorine-based disinfectants. In the laboratory, aqueous solutions containing 2–2.5% H_2_SO_4_ allow the storage of oocysts for at least 1 year without affecting significantly either their structure or infectivity (Dubey et al., 1970a, Speer et al., 1998). To a lesser extent, the sporocyst wall by itself can withstand acidic degradation, as demonstrated by free sporocysts stored for 20 days in 2% H_2_SO_4_ solution retaining infectivity for laboratory mice (Christie, 1977). Interestingly, in vitro experiments suggest that the walls of oocysts engulfed by free-living amoeba can resist the intracellular acid environment and remain infectious to laboratory mice (Winiecka-Krusnell et al., 2009). In vivo, oocysts appear to overcome acidic conditions found in the stomach to safely reach the intestine where they are thought to release their sporozoites. This later point is discussed in further detail in Section “In infection of the host”.

Chlorinated disinfectants cause more visible modifications of the oocyst wall without killing the sporozoites. Indeed, *T. gondii* oocysts can remain infective following incubation with bleach, free chlorine, chlorine dioxide, and chloramine at concentrations and exposure times corresponding to typical household or industrial uses (Villena et al., 2002, Wainwright et al., 2007b, Villegas et al., 2010). Exposing oocysts to household bleach solutions containing 3–10% sodium hypochlorite (NaOCl) for 30 min leads to the detachment and dissolution of the outer layer of the oocyst wall but does not affect neither the structure nor the permeability of the inner layer of the oocyst wall and the sporocyst wall (Dubey et al., 1970b, Dumètre et al., 2013). Similarly, bleach treatment does not modify significantly the mechanical characteristics of the oocyst wall. The inner layer appears to be as rigid and robust as the non-modified bilayered oocyst wall as tested by indentation using atomic force microscopy (AFM) (Dumètre et al., 2013). Comparing the oocyst wall proteomes of bleach-treated vs. untreated oocysts indicates that some of structural proteins, such as the PAN-domain containing proteins, are no longer detected in bleach-treated oocysts (Fritz et al., 2012a). Moreover, bleach-treated oocysts are significantly less autofluorescent than untreated oocysts (Dumètre et al., 2013), which suggests that notable amount of tyrosine-rich proteins forming dityrosine bonds can be deleted following bleach exposure. The removal of the outer most oocyst wall layer will lead to the exposure of molecules of the inner layer at the oocyst surface in particular beta 1–3 glucans. Interestingly, beta 1–3 glucans in yeast cell wall have been shown to be quite resistant to oxidation by NaOCl (Ohno et al., 1999).

In the light of these studies, *T. gondii* oocysts could resist chlorinated agents due to the structure and molecules of their inner oocyst wall layer. In particular, beta 1–3 glucans deserve further attentions as they are proposed to withstand chlorine and organic solvents in yeasts and *E. tenella* oocysts (Bushkin et al., 2012, Bushkin et al., 2013). The resistance of the sporocyst wall to chemical agents is less well documented. It appears as a second protective barrier for the sporozoites in case of a complete disruption of the oocyst wall and potentially possesses different mechanical behaviours due to the sutures at its inner layer. The sporocyst wall may also have an important role during the process of the parasite’s infection within the host digestive tract.

### In the dissemination of the parasite from land to sea

The transport dynamics of oocysts in soils and waters is governed by parasite as well as abiotic and biotic environmental factors. Parasite factors include oocyst size, specific gravity, shape and surface properties of the oocyst wall (such as electric charges and hydrophobicity) (Dumètre et al., 2012). Abiotic environmental factors include the physicochemical properties of soils and waters in which oocysts are located, in conjunction with climatic events (Dumètre et al., 2012). Biotic environmental factors include plants (Kniel et al., 2002), algae, and invertebrates such as earthworms (Ruiz and Frenkel, 1980) and filter-feeding molluscs (VanWormer et al., 2013) that can retain infective oocysts at their surface or within their tissues upon filtration.

Insights on the surface properties of *T. gondii* oocysts may help unravel the parasite’s environmental transmission mechanisms, which in turn may impact on delivery of *T. gondii* to susceptible hosts. The bilayered wall of freshly excreted and purified oocysts is negatively charged in low-ionic strength solutions (zeta potential (ζ) of −43.7 and −16.16 mV in ultrapure water and artificial freshwater, respectively, and hydrophilic) (Shapiro et al., 2009). In addition, oocysts were found to be faintly non-specifically 'sticky' using AFM (Dumètre et al., 2013) but do not adhere to each other even after forced contact for longer than a minute (Dumètre, A., and Puech, P.-H., personal observations). Under natural settings, oocysts are in contact with various organic and inorganic compounds that may affect these properties. Addition of organic matter to oocysts in freshwater solutions tends to increase their negative charges in a concentration-dependent manner (ζ_max_ = −21.09 mV) (Shapiro et al., 2009). The negative charge of oocysts implies that electric repulsion forces lead to diminished interactions between oocysts and other surfaces, in turn favouring the mobilization of the parasites from faecal deposits to soils and, following rainfall, to surface waters. This process results in the progressive transport and dilution of the oocyst load across time and space. Conversely, diminished surface charge in higher ionic strength waters favours adhesion or physical entrapment of the oocysts, thus promoting their retention and concentration at a given point. In solutions mimicking the estuarine and marine environments, the oocyst surface charge approaches neutral (ζ = −1.84 and −2.81 mV, respectively) because of cations decreasing the oocyst surface negative charges (Shapiro et al., 2009). These findings suggest that oocysts are prone to disperse in freshwaters and soils having low cation exchange capacity (i.e. low adsorption capacity) while they tend to aggregate to each other and to other particles and surfaces they may encounter in presence of dissolved cations, as in coastal and marine environments (Dumètre et al., 2012).

Advances in the characterization of oocyst surface properties have further yielded new insight on the mechanisms that mediate *T. gondii* exposure to marine mammals, where infections have been particularly puzzling due to the strictly terrestrial nature of the parasite’s definitive hosts (felids). Specifically, the reduced oocyst wall charge and enhanced particle interactions in higher salinity waters results in increasing incorporation of *T. gondii* oocysts into organic marine aggregates (aka ‘marine snow’) (Shapiro et al., 2012). Reduced repulsive forces are also likely to play a role in the direct adhesion of oocysts to sticky biofilms such as those that coat kelp in many coastal regions (Mazzillo et al., 2013). Combined, the association of oocysts with sinking aggregates and sticky emergent vegetation or algae lead to retention and concentration of *T. gondii* in coastal zones that receive freshwater runoff – leading to high-risk infection zones to marine animals that utilize these habitats. The transformation of waterborne oocysts into benthic particles via settling of aggregates or entrapment in biofilms further mediate transmission to susceptible hosts by enabling the incorporation of *T. gondii* into marine food webs, e.g. through benthic-grazing invertebrates. Specifically, kelp-grazing snails have been shown to effectively capture and retain *T. gondii* oocysts, thereby delivering oocysts to predators such as southern sea otters that suffer mortality due to toxoplasmosis (Shapiro et al., 2014). The robust nature of oocyst walls likely aids in the persistence of the parasite in passage through invertebrate gut – as demonstrated by the presence of brightly autofluorescing oocysts visualized in faeces excreted by snails up to 11 days following exposure (Krusor et al., 2015).

In conclusion, the physicochemical properties of the oocyst wall in conjunction with environmental conditions are critical factors that mediate oocyst transmission to different host species living in diverse biotopes. Additional studies are required to better characterize the wall properties of oocysts over time and following exposure to chemical disinfectants as applied in food and water industries. Some preliminary studies indicate that aged oocysts tend to aggregate (Dumètre and Dardé, 2004) and that oocysts exposed to chlorinated disinfectants could be more 'sticky' than untreated oocysts (Dumètre et al., 2013) because of the modifications of the structure and molecular composition of their wall (Dumètre et al., 2013, Harito et al., 2016). This behaviour could favour oocyst aggregation and subsequent host exposure to higher inoculum doses of aged but still infective oocysts in the environment.

### In infection of the host

Following oocyst ingestion, the sporozoites have to excyst from the oocyst and sporocyst walls before invading host cells and differentiating into rapidly replicating tachyzoites. In laboratory mice, sporozoites were found almost exclusively in the ileal enterocytes as early as 30 min after feeding oocysts (Dubey et al., 1997b). However, the release of the sporozoites from the oocyst has never been observed in vivo. In vitro, excystation can only be achieved by using harsh physical methods (Movie S1) and/or chemical stimuli (Movie S2) (Christie et al., 1978, Dubey et al., 1970b, Ferguson et al., 1979d, Freyre and Falcón, 2004), which partly mimic the microenvironment of the host digestive tract.

The first step of excystation requires the opening of the oocyst wall but how this occurs in vivo, given its robust nature, is unclear (Movie S1) (Dumètre et al., 2013). Though acidified pepsin solutions can affect the oocyst wall surface, for instance by unmasking WGA-reactive glycoproteins (Harito et al., 2016), digestive-like solutions containing pepsin/HCl, proteolytic agents, trypsin or bile salt mixture (e.g. sodium choleate) do not significantly affect the macroscopic integrity of the oocyst wall when used separately or in combination at physiological temperature and pH (Christie, 1977, Christie et al., 1978, Dubey et al., 1970b, Ferguson et al., 1979d, McKenna and Charleston, 1982, Freyre and Falcón, 2004). The oocyst wall can be weakened or ruptured in vitro using sonication or grinding to cause mechanical disruption, possibly under anaerobic conditions (Ferguson et al., 1979d, Freyre and Falcón, 2004). Such methods can result in the releasing of intact sporocysts from the damaged oocyst wall. In vitro, previous works on oocysts of some *Eimeria* (Jensen et al., 1976, Jolley et al., 1976) and *Cystoisospora* (*Isospora*) species (McKenna and Charleston, 1982) with or without a micropyle suggest that dissociated CO_2_ in combination with cysteine hydrochloride as a reducing agent increases the permeability of the oocyst wall and progressively alters its integrity by enhancing the reduction of disulfide bridges of structural wall proteins. In *T. gondii*, proteins that forms disulfide bridges in the oocyst wall, such as the members of the TgOWP family (Possenti et al., 2010), could be triggered by the combined action of CO_2_ and reducing agents (yet to be identified in vivo). Alternatively or concomitantly, rapid pH variation (from 1.5 in stomach to 7.8 in duodenum) could also contribute to cracking the oocyst wall as suggested in models of multi-layered microcapsules (Lulevich and Vinogradova, 2004).

**Fig. 7:**
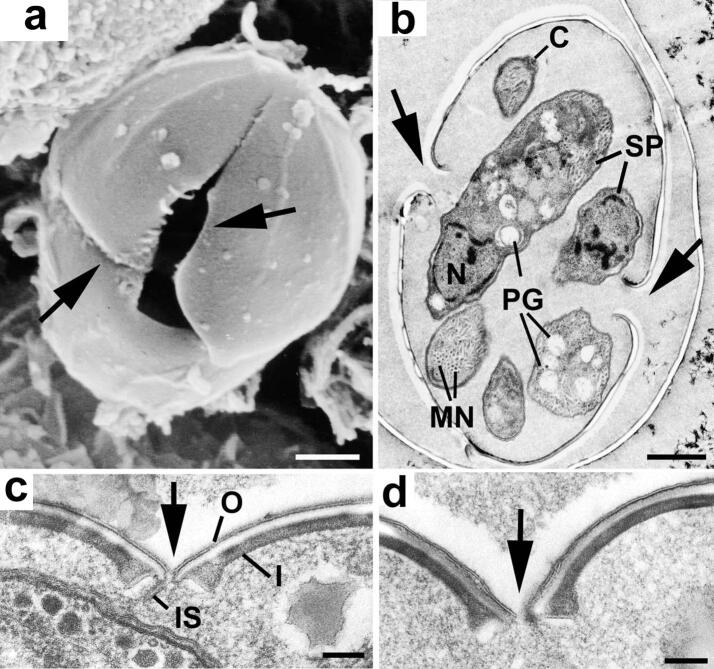
Electron micrographs showing changes in the sporocyst wall associated with excystation. (a) Scanning electron micrograph of a sporocyst undergoing excystation, showing the separation of the plates of the sporocyst wall (arrows). Bar = 1 µm. (b) Transmission electron micrograph through an excysting sporocyst, showing separation and in folding of the plates of the sporocyst wall (arrows). The sporozoites (SP) contain a posteriorly located nucleus (N), numerous micronemes (MN) and polysaccharide granules (PG). C, conoid. Bar = 1 µm. (c) Early stage in excystation showing inward curling (arrow) of the sporocyst wall at the junction of the plates of the inner layer (I), resulting in a separation of the plates and the intermediate strip (IS). O, outer layer. Bar = 100 nm. (d) More advanced stage of excystation showing separation of the inner plates and rupture of the outer layer (arrows). Bar = 200 nm. The techniques used and images are from Ferguson et al. (1979d) with permission.

The second step of the sporozoite excystation consists in the opening of the sporocyst wall and the subsequent release of the sporozoites. Following weakening or opening of the oocyst wall, the wall of the sporocysts is thought to become more sensitive to the detergent action of bile salts, in particular at the intermediate strips that maintain the curved plates forming the inner layer of the sporocyst wall of *T. gondii* (Fig. 7) (Christie et al., 1978, Ferguson et al., 1979d, Speer et al., 1998) and of closely-related *Isospora* species (McKenna and Charleston, 1982). Upon bile treatment, the sporozoites start moving inside the microscopically intact sporocysts, either free from or still enclosed by the oocyst wall (Movies S3 and S4 respectively) (Christie et al., 1978, McKenna and Charleston, 1982). A decrease in the strength of the sporocyst wall at the plate junctions coupled to the rapid movements of the sporozoites results in splitting of the plates, which appear to be under tension and curl inwards, allowing the sporozoites and the internal residual mass to be released from the sporocyst (Fig. 7 and Movie S3) and the oocyst walls (Movie S5) (Christie et al., 1978, Ferguson et al., 1979d). It appears that oocyst and sporocyst walls retain their autofluorescence during the excystation process (Fig. S1). In vivo, free sporozoites immediately invade the nearby enterocytes and then differentiate into tachyzoites within 24 h.

To sum up, opening and/or degeneration of the *T. gondii* oocyst walls in the small intestine could stem from the combined action of dissolved CO_2_, pH variations and a cocktail of digestive agents the oocysts experience from mouth to intestine. If contact between the host digestive microenvironment and the oocyst is required for priming the infection under natural settings, it appears however dispensable for opening the oocyst walls, at least in vitro. Indeed, macrophage cells can sustain sporozoite excystation following oocyst internalisation suggesting that the macrophage molecular content could also lead to oocyst wall opening (Freppel et al., 2016).

## Conclusion and perspectives: targeting the oocyst walls' molecules and structure

From terrestrial to aquatic environments, *T. gondii* oocyst walls confer great resistance to various conditions and inactivation procedures. Beyond their protective role, the oocyst walls have been recently shown to be interactive surfaces that can affect the transmission of the parasites from soils, waters, and foodstuffs to tissues of numerous host species. It is still unclear how long the wall of oocysts retains its bilayered structure and integrity under diverse environmental conditions. Partial degradation of the outermost wall layer due to oocyst age and/or exposure to chemical stresses does not preclude sporozoite infectivity and could favour the retention of oocysts in soils, water sediments, at the surface of vegetables and in the tissues of transport hosts. Future studies should investigate the effects of different chemical and physical stresses on the structure and molecules of the oocyst wall over time, and their consequences on the transmission and survival of oocysts from land to sea. Whether wall modifications enhance or reduce host infection also require further investigations exploring the normal and perturbed permeability of the oocyst and sporocyst walls, which is currently unknown.

In a more applied perspective, targeting the structure and molecules of the oocyst walls could aid detection of oocysts in diverse environmental matrices, and their potential elimination. For instance, the recent identification of a WGA-reactive glycoprotein in the outer oocyst wall layer has led to the development of a lectin-magnetic separation assay for isolating oocysts in environmental waters (Harito et al., 2016, Harito et al., 2017). However, research on *T. gondii* oocysts remains challenging due to a lack of in vitro oocyst producing system and the biohazard risks associated with oocyst manipulation, oocysts being highly infectious to humans. Instead, other coccidian parasites not infectious to humans (Kniel et al., 2002, Mai et al., 2009) or particles with similar surface properties (Shapiro et al., 2009) are increasingly considered as surrogates for *T. gondii* oocysts in transport or disinfection experiments. Such surrogate models could be very valuable for modelling oocyst transmission and developing appropriate strategies to limit environmental contamination and reduce subsequent human and animal infections.

## Figures and Tables

**Supplementary Figure S1:**