# Supplementary material for: Structure, composition, and roles of the Toxoplasma gondii oocyst and sporocyst walls
Source: Cell Surf. 2018 Dec 19;5:100016. doi: 10.1016/j.tcsw.2018.100016 (PMC7389338; doi:10.1016/j.tcsw.2018.100016)
Supplement: Supplementary data 8 [file mmc8.docx]

**SUPPLEMENTARY INFORMATION**

**Title:** Structure, composition, and roles of the *Toxoplasma gondii* oocyst and sporocyst walls

**Table S1: Reagents for highlighting the oocyst and sporocyst walls of *Toxoplasma gondii*.**

**Movie S1: Time-lapse video microscopy showing mechanical indentation and rupture of the walls of a *T. gondii* oocyst by using a glass needle.** Oocyst suspended in RPMI medium was aspirated and held with a micropipette under aspiration at controlled pressure (5 cm H_2_O) (see Freppel et al., 2016 for methodology). Then, an home-made glass needle was pressed on the oocyst wall surface first with moderate forces, leading to deformations and “snap-back” reminiscent of table tennis ball ones, then with higher forces until the oocyst and sporocyst walls ruptured. Scale bar = 5 µm. Movie runs at 20 frames per second.

**Movie S2: Time-lapse video microscopy of *T. gondii* oocysts incubated in excystation fluid.** Three-month old oocysts of the VEG strain were prepared according to the method described by Freppel et al. (2016). Briefly, oocysts in 100 µl Hanks' balanced salt solution (HBSS) were submitted to low powerful sonication, a method that usually results in a mixture of microscopically intact oocysts and few free sporocysts (arrows). Then, oocysts were transferred in a 35-mm Petri dish containing 3 ml pre-warmed HBSS that was placed on a microscope (Zeiss Axiovert 200, equipped with a 20x lens and a CoolSnap HQ2 camera) equipped with a chamber heated at 37°C (JPK PetriDish Heater, JPK Instrument). Following oocyst sedimentation and temperature stabilization for 20 min, 600 µl of 5X pre-warmed excystation fluid containing sodium choleate and sodium bicarbonate in HBSS were introduced in oocyst suspension to induce sporozoite excystation. Note that sporozoites excysted more easily from free sporocysts (arrows, see details in Movie S2) than within oocysts (see details in Movie S3). The movie starts at t=123 sec, i.e. 123 sec after adding the excystation fluid. Time interval is 1 sec except between frames 172 and 173 (10 sec). Scale bar is 10 µm. Movie runs at 10 frames per second.

**Movie S3: Time-lapse video microscopy of *Toxoplasma gondii* sporozoites excysting from a free sporocyst captured in Movie S2.** Note the increasing movements of the sporozoites within the sporocyst before excystation at ~480 sec. The movie starts at t=123 sec, i.e. 123 sec after adding the excystation fluid. Time interval is 1 sec except between frames 172 and 173 (10 sec). Scale bar is 10 µm. Movie runs at 10 frames per second.

**Movie S4: Time-lapse video microscopy of *Toxoplasma gondii* sporozoites excysting within an oocyst captured in Movie S2.** Note the increasing movements of the sporozoites within one of the two sporocysts (arrow) before they excyst at ~280 sec. The movie starts at t=123 sec, i.e. 123 sec after adding the excystation fluid. Time interval is 1 sec except between frames 172 and 173 (10 sec). Scale bar is 10 µm. Movie runs at 10 frames per second.

**Movie S5: Time-lapse microscopy of *Toxoplasma gondii* sporozoites excysting through a gap in the wall of an oocyst.** The movie starts at t=525 sec, i.e. 525 sec after adding the excystation fluid. Scale bar is 10 µm. Movie runs at 10 frames per second.

**Figure S1: Representative morphology of oocysts and sporocysts of *Toxoplasma gondii* after 20 min in excystation fluid.** Oocysts were submitted to excystation fluid as described in Movie S2 legend prior to be observed under bright field (left) or UV excitation for recording the autofluorescence pattern of the oocyst and sporocyst walls (right). Most of oocysts contain wall remnants of at least one sporocyst (white arrowheads). Wall remnants of a sporocyst are also visible (yellow arrowhead). Note that sporozoites are not autofluorescent under UV excitation (red arrowheads). Scale bar = 10 μm.

**
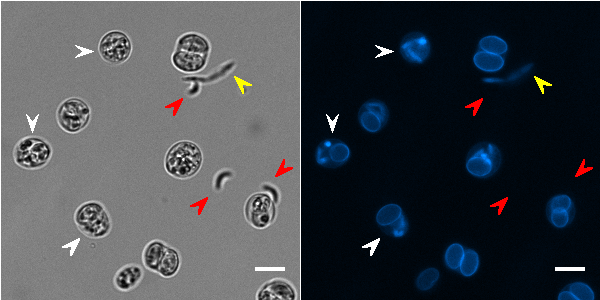
**

**References**

Freppel, W., Puech, P.-H., Ferguson, D.J.P., Azas, N., Dubey, J.P., Dumètre, A., 2016. Macrophages facilitate the excystation and differentiation of *Toxoplasma gondii* sporozoites into tachyzoites following oocyst internalisation. Sci Rep 6, 33654. https://doi.org/10.1038/srep33654
